# The Role of IgE in Upper and Lower Airway Disease: More Than Just Allergy!

**DOI:** 10.1007/s12016-021-08901-1

**Published:** 2021-09-18

**Authors:** Philippe Gevaert, Kit Wong, Lauren A. Millette, Tara F. Carr

**Affiliations:** 1 Upper Airway Research Laboratory, Department of Otorhinolaryngology, Ghent University Hospital, Ghent, Belgium; 2 Genentech, Inc., 1 DNA Way, CA 94080 South San Francisco, USA; 3 Asthma and Airway Disease Research Center, University of Arizona, Tucson, AZ USA

**Keywords:** Asthma, Immunoglobulin E, Lower airway disease, Nasal polyps, Rhinitis, Upper airway disease

## Abstract

Immunoglobulin E (IgE) is a well-known key factor in allergic airway disease; however, its central role in non-allergic airway inflammation is often underestimated. In some airway diseases, IgE is produced as a result of allergic sensitization. However, in others, IgE production occurs despite the lack of a specific allergen. Although multiple pathways contribute to the production of IgE in airway disease, it is its activity in mediating the inflammatory response that is associated with disease. Therefore, an understanding of IgE as the unifying component of upper and lower airway diseases has important implications for both diagnosis and treatment. Understanding the role of IgE in each upper and lower airway disease highlights its potential utility as a diagnostic marker and therapeutic target. Further classification of these diseases by whether they are IgE mediated or non–IgE mediated, rather than by the existence of an underlying allergic component, accounts for both systemic and localized IgE activity. Improvements in diagnostic methodologies and standardization of clinical practices with this classification in mind can help identify patients with IgE-mediated diseases. In doing so, this group of patients can receive optimal care through targeted anti-IgE therapeutics, which have already demonstrated efficacy across numerous IgE-mediated upper and lower airway diseases.

## Introduction

Immunoglobulin E (IgE) is widely accepted as an integral component of the pathogenesis of many allergic respiratory diseases, with increasing recognition of involvement in the non-allergic forms of disease. This is evident in both upper airway diseases, including allergic rhinitis (AR), non-allergic rhinitis (NAR), and chronic rhinosinusitis with nasal polyps (CRSwNP), and lower airway diseases, including allergic asthma and non-allergic asthma phenotypes (eosinophilic asthma, aspirin-exacerbated respiratory disease [AERD], and non-allergic occupational asthma) [1].

The shared aspects of otherwise discrete airway diseases have led to a continuous or “unified airway” concept for which pathological processes of the upper airway are thought to mirror lower airway events [2, 3]. The primary pathological process in both upper and lower airway disease is driven by inflammation, which can be caused by both allergic (immune response triggered by exposure to an allergen) and non-allergic (triggered by exposure to an environmental factor, resulting in activation of the innate immune pathway) disease mechanisms [4–6]. In support of this notion, upper and lower airway diseases often coexist [3, 7] and share common inflammatory processes, including those mediated by IgE [2, 8–10]. There are multiple types of IgE that have been linked to airway inflammation: specific IgE antibodies that are produced in response to allergen exposure, and non-specific IgE antibodies that are produced following the activation of the body’s innate immune system or through the action of superantigens [5, 6]. IgE is routinely described in the pathology of both acute and chronic inflammatory allergic diseases [11], and elevated IgE levels are also commonly reported in non-allergic late-onset asthma and nasal polyps (NP), in which the emergence of an IgE response need not be specific [10, 12]. In some cases, *Staphylococcus aureus*–derived enterotoxins have been proposed to act as superantigens in patients with NP or asthma to produce a local polyclonal IgE response [10, 13].

Historically, the term “allergy” was used to describe the organ-specific or systemic antigen-specific IgE-mediated immune response of the skin and mucosa in response to extrinsic allergen exposures [14]. However, as knowledge of disease pathology has increased, it has become clear that IgE-mediated disease exists regardless of allergen exposure. In NAR, symptomology is identical to AR, despite a negative skin prick test (SPT) for allergic response [14]. Similarly, between 10 and 33% of patients with asthma have an intrinsic, non-allergic form associated with a later, more severe presentation than allergic asthma [14]. It has also been shown that NAR in childhood is associated with a significant risk of developing asthma in adulthood, highlighting the need for exploring non-allergic components of disease [15]. In CRSwNP, IgE plays a major role in disease pathology, which is dominated by T helper 2 (Th2) inflammatory patterns [1, 16]. Although local IgE produced in the NP tissue can be the result of allergic stimulation, NP are also found in patients with no allergic sensitization. Here, the local IgE produced is polyclonal and functional, often as a result of the innate immune response or through the actions of *S. aureus* enterotoxins [1]. The underlying pathologies of allergic and non-allergic asthma are considerably different. Allergic asthma is defined as elevated systemic specific IgE and/or a positive SPT following an allergen exposure, and non-allergic asthma shows no such response to routine allergy tests [17]. In these patients with non-allergic asthma, production of IgE and local tissue IgE or eosinophil level drive disease, which typically occurs later in life and with more severe presentation compared with allergic asthma [17]. However, despite the lack of allergic sensitization, many diseases of the upper and lower airways are under the influence of local IgE activity [1].

Therefore, applying the learnings surrounding the role of IgE in upper and lower airway diseases in the context of both allergic and non-allergic phenotypes has important implications for treatment.

## IgE-Mediated Disease

**Fig. 1:**
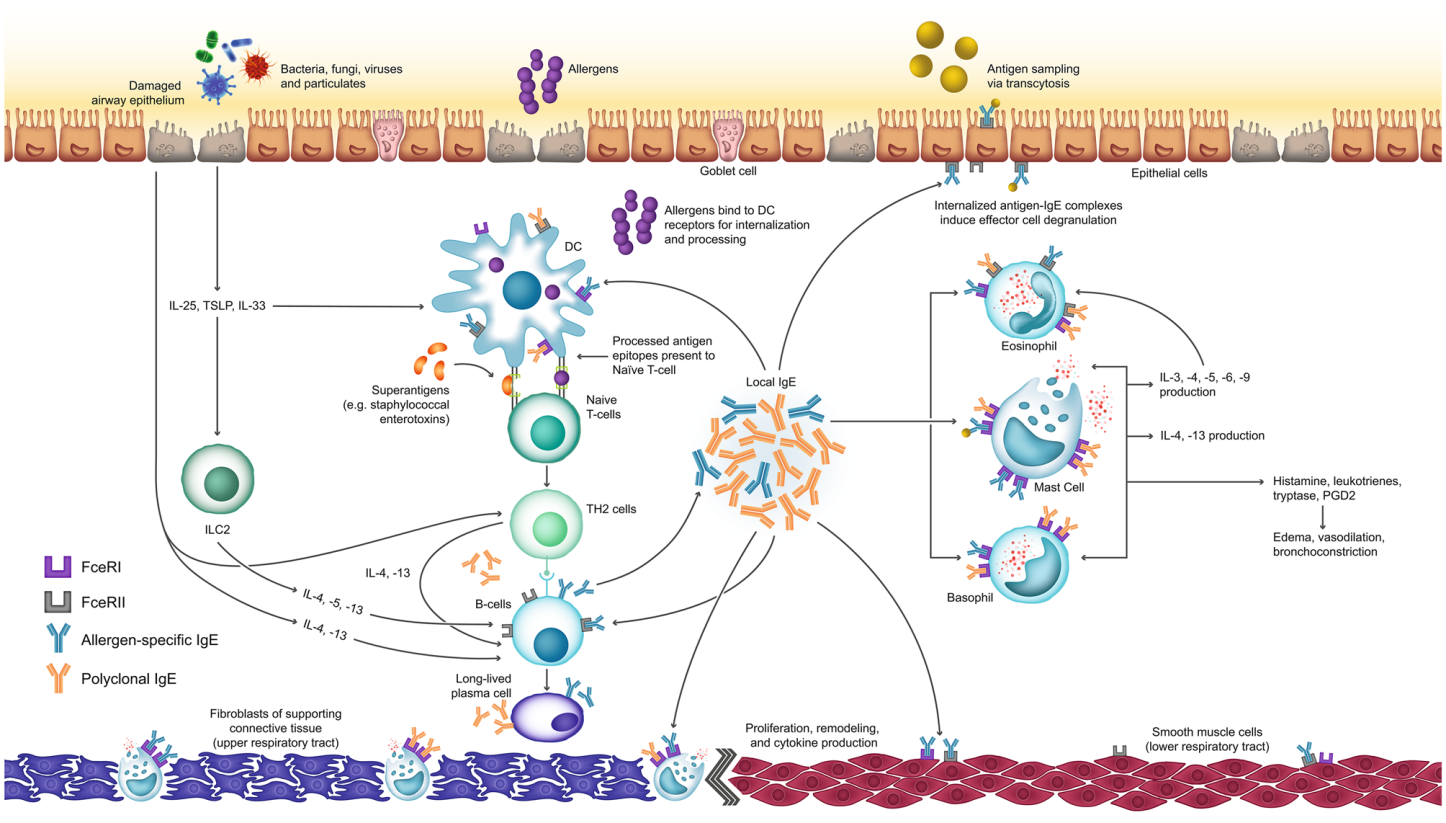
Mechanism of IgE-mediated upper and lower airway disease. In response to allergen exposure, dendritic cells present allergen-specific antigens to naïve T cells, which are activated and differentiate into Th2 cells. These Th2 cells produce key cytokines (IL-4, IL-13), prompting B cells to produce allergen-specific IgE. Alternatively, exposure to external stimuli such as bacteria, fungi, viruses, and particulates promotes epithelial release of IL-25, TSLP, and IL-33. These factors stimulate ILC2 cells to produce IL-5, IL-13, and to a lesser extent, IL-4, which in turn promote B cell production of IgE. Finally, superantigens, including *Staphylococcal* enterotoxins, can directly cross-link antigen-presenting cells with naïve T cells, bypassing the antigen presentation step, yielding polyclonal IgE. Once produced, local IgE acts on the FcεRI receptors of tissue-resident mast cells and basophils, prompting the release of histamine, leukotrienes, tryptase, and prostaglandin, which manifest as edema, vasodilation, and bronchoconstriction as part of the early response. IgE also binds to FcεRII receptors on B cells for enhanced antigen presentation. Later release of key cytokines recruits proinflammatory cells, including eosinophils and basophils, to the site of inflammation, and additionally promotes the overexpression of mucus-producing goblet cells and contributes to airway hyperresponsiveness. Crosstalk within the inflammatory pathway promotes a self-propagating cycle of chronic inflammation. The lower left side of the figure depicts the fibroblasts and mast cells within the supporting connective tissue of the nasal cavity, while the lower right side depicts the smooth muscle cell layer surrounding the lower respiratory airway. *DC*, dendritic cell; *FcεRI*, high-affinity immunoglobulin E receptor; *FcεRII*, low-affinity immunoglobulin E receptor; *IgE*, immunoglobulin E; *IL*, interleukin; *ILC*, innate lymphoid cell; *PGD2*, prostaglandin D2; *Th2*, T helper 2; *TSLP*, thymic stromal lymphopoietin

Many diseases of the upper and lower respiratory systems share a common underlying mechanism in the type 2 inflammatory pathway. This pathway is mediated by several key cell types, including eosinophils, mast cells, basophils, Th2 cells, type 2 innate lymphoid cells (ILC2), and IgE-producing B cells. Elevated IgE is a hallmark of type 2 inflammation, where it plays a key effector role in the propagation of the immune response [18]. For simplification, the IgE-mediated process can be broken down into two phases: IgE production and effects of IgE signaling (Fig. 1).

### IgE Production

Evolutionarily, IgE contributes to the body’s defense against parasites such as helminths [5, 19]. However, environments favoring type 2 inflammation, such as acute and chronic exposures to factors including allergens, bacteria, fungi, viruses, and particulates can lead to B cell class switching and IgE production, which mediates the inflammatory cascade considered to be a major factor in many diseases of the upper and lower airways [1, 11, 13, 20]. B cells are responsible for the generation of these powerful immune responses through the production of a variety of Ig isotypes, including IgE, based on the location and type of exposure [21, 22].

**Fig. 2:**
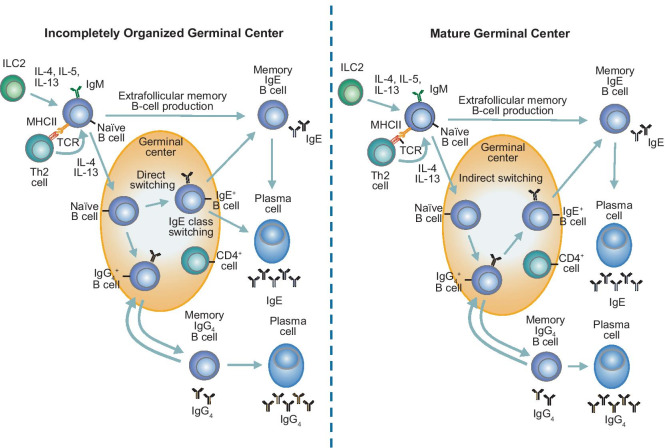
B cell activation. In response to Th2-derived IL-4 and IL-13, naïve B cells migrate to B cell follicles for proliferation and formation of germinal centers. In incompletely organized germinal centers, as found in the Th2-centric response, naïve B cells undergo somatic hypermutation and class-switch recombination as part of direct switching to IgE^+^ B cells. In mature germinal centers, naïve B cells undergo indirect switching, passing through an intermediate IgG_4_^+^ B cell phase before transforming into IgE^+^ B cells. In either case, IgE^+^ B cells can then leave the germinal center, becoming either memory B cells or long-lived plasma cells. Memory B cells are dividing cells that produce minimal IgE but allow the prompt production of specific IgE-secreting plasma cells following a secondary allergen exposure in the absence of cytokines. It is not well established where IgE memory resides, and this remains a topic under active investigation within the field. Plasma cells do not divide, but produce far more specific IgE. Figure adapted from Akdis M and Akdis CA. Nat Immunol 2012;13(4):312–314 and Davies JM, et al. J Allergy Clin Immunol 2013;131(4):972–976. *IgE* immunoglobulin E, *IgG* immunglobulin G, *IgM* immunoglobulin M, *IL* interleukin, *ILC *innate lymphoid cell, *MHCII* major histocompatibility complex class II, *TCR* T cell antigen receptor, *Th2* T helper 2

In a localized allergic response, after an initial allergen exposure, antigen-presenting dendritic cells sensitize naïve T cells, promoting their development into Th2 cells. These cells produce the inflammatory cytokines interleukin (IL)-4 and IL-13, leading to B cell activation and production of specific IgE (Fig. 2) [22, 23].

Meanwhile, in a systemic allergic response, B cells are exposed to antigens in peripheral lymphoid organs, and can then move either into extrafollicular areas for proliferation and differentiation into short-lived plasma cells, or into B cell follicles for proliferation and formation of germinal centers [22, 24]. In the germinal centers, activated B cells undergo genetic modifications to the Ig gene (somatic hypermutation and class-switch recombination), resulting in the production of different Ig isotypes that are highly specific for the antigen [22, 25, 26].

B cells primarily produce two Ig isotypes in response to an allergen exposure: IgE and IgG4. IgE production occurs early, before IgG4 levels rise, though after frequent exposures, IgG4 levels may also rise [26]. Once B cells switch to IgE production and leave the germinal center, they become either memory B cells or long-lived plasma cells. Memory B cells are dividing cells, which only secrete a small amount of IgE. Following a secondary exposure, however, memory B cells allow the prompt production of specific IgE–secreting plasma cells. Conversely, long-lived plasma cells do not divide but produce far more specific IgE [1].

More recent evidence has shown that high levels of local IgE and IgE-producing B cells are also present in the nasal mucosa and lungs of patients regardless of allergy status, suggesting that the previously described germinal center reactions (somatic hypermutation and class-switch recombination) can also occur in the local tissue [5, 22, 25, 27].

In healthy individuals, the respiratory epithelium serves as a physical barrier to protect the upper and lower airways from environmental exposures, including viruses, bacteria, fungi, allergens, and contaminants [28]. Epithelial dysfunction due to loss of E-cadherin– and claudin-mediated epithelial tight junctions [24, 28] exposes the airways to these environmental factors, triggering an inflammatory response through the innate immunity pathway, which has been reported in many upper and lower airway diseases, including CRSwNP, asthma, and rhinitis [5, 13, 28]. Epithelial cells are also capable of detecting these exposures through pattern recognition receptors, triggering the release of key mediators, termed “alarmins,” including thymic stromal lymphopoietin (TSLP), IL-25, and IL-33, in response to exposure [24]. These signaling molecules cause ILC2 cells to proliferate and produce IL-5 and IL-13 and, to a lesser extent, IL-4 [29]. Of note, ILC2 cells produce approximately ten-fold more IL-5 and IL-13 compared with activated Th2 cells, and therefore play a vital role in the propagation of the type 2 inflammatory response [24]. ILC2- and Th2-derived IL-5 promotes the differentiation, maturation, mobilization, and survival of eosinophils and supports the development of mast cells and basophils, amplifying the inflammatory response [24]. The alarmins, TSLP, IL-25, and IL-33, can also stimulate dendritic cells to induce naïve T cells into IL-4– and IL-13–producing Th2 cells, further driving the immune response [29] through B cell activation and revision, and the production of IgE [22, 23]. Meanwhile, Th2-derived IL-2 stimulates ILC2 cells, resulting in a positive feedback loop promoting inflammation [29].

Additionally, disruption of the epithelial barrier can allow the entry of bacterial proteins, which can act as superantigens, into the bronchial tissue, leading to a shift toward Th2-mediated inflammation, with increases in eosinophilia, IL-5, and IgE [1, 30]. Superantigens hijack the body’s T cell antigen recognition system by directly cross-linking major histocompatibility complex class II on antigen-presenting cells and T cell receptors on T cells, bypassing the conventional antigen presentation step, and leading to a powerful immune response [30]. This promotes the production of polyclonal IgE, in contrast to the monoclonal IgE produced by the allergic and innate immune responses. Of note, polyclonal IgE may mask the effects of specific IgE by overwhelming the receptors on mast cells [25].

Regardless of whether IgE is produced through allergic sensitization, activation of the innate immune system, or by exposure to superantigens, these pathways are known to converge in the IgE-mediated inflammatory signaling cascade.

### Effects of IgE Signaling

IgE is fundamental in the type 2 inflammatory response through its interaction with its two receptors: the high-affinity IgE receptor (FcεRI) and the low-affinity IgE receptor (FcεRII), also referred to as CD23 [24]. IgE has been implicated in a positive feedback mechanism wherein it contributes to the upregulation of both FcεRI and FcεRII, leading to an enhanced hypersensitivity response [31].

Secreted specific IgE binds to FcεRI on mast cells and basophils, sensitizing them to specific allergens ahead of future exposures [23]. Subsequent exposure to an allergen leads to the cross-linking of membrane-bound IgE in mast cells and basophils, inducing cellular degranulation and release of histamine, tryptase, cysteinyl leukotrienes, and platelet-activating factors [23]. The actions of these early response factors can manifest as edema, vasodilation, and bronchoconstriction (Fig. 1) [23].

Following these early events, the later release of cytokines and chemokines (e.g., IL-3, IL-4, IL-5, IL-13, CC chemokine ligand 5, and granulocyte–macrophage colony-stimulating factor) from mast cells and basophils recruits inflammatory cells, including eosinophils, to the site of inflammation (Fig. 1) [23]. Released IL-4 and IL-13 promote the overexpression of mucus-producing goblet cells and contribute to airway hyperresponsiveness [24]. Basophil-derived IL-4 has been shown to directly enhance the function of ILC2 [24]. Importantly, the cross-linking of IgE to FcεRI on mast cells and the subsequent release of IL-4 aids in Th2 differentiation and B cell isotype switching, leading to the production of additional IgE in a positive feedback loop, which can lead to chronic inflammation [24].

Meanwhile, IL-5 promotes the differentiation, maturation, mobilization, and survival of eosinophils and supports the development of mast cells and basophils [24]. The recruitment of eosinophils to sites of inflammation is largely dependent on coordination between IL-5, eotaxin, and IL-13, wherein IL-13 recruits eosinophils to specific tissues under the regulation of IL-5 and eotaxin [32]. Once recruited, eosinophils induce epithelial damage through accumulation, degranulation, and release of toxic proteins, including eosinophil-derived neurotoxin, eosinophil cationic protein, eosinophil peroxidase, and major basic protein [33]. Eosinophils also serve as a source of proinflammatory cytokines (IL-3, IL-4, IL-6, granulocyte–macrophage colony-stimulating factor, tumor necrosis factor α, transforming growth factor β) and chemokines and are able to present antigens to T cells, further propagating the inflammatory cascade [33].

Beyond the expression of FcεRI on mast cells and basophils, its constitutive expression on dendritic cells also plays an important role in the type 2 inflammatory pathway [34, 35]. The role of antigen presentation by dendritic cells is closely linked to the cross-linking of IgE and FcεRI. Studies have shown that FcεRI aids in the presentation of IgE-bound antigens, including allergens, to T cells. These bound antigens are internalized, processed, and presented to T cells, leading to T cell maturation and continuation of the type 2 inflammatory cascade [35]. Of note, signaling following dendritic cell IgE/FcεRI cross-linking has been implicated in the mediation of type 2 inflammation in either a proinflammatory or suppressive manner, depending on the cytokines produced [35].

Another important role of FcεRI in airway disease involves its expression in airway smooth muscle tissue. This tissue has been shown to respond directly to environmental exposures, including dust, microbes, and pollutants, in addition to intrinsic cytokines and Ig [36]. Exposure to IgE, whether it be antigen-bound IgE or independent, and subsequent cross-linking with FcεRI on airway smooth muscle cells, leads to the release of the proinflammatory cytokines IL-4, IL-5, and IL-13, as well as chemokines CC chemokine ligand 11/eotaxin [36].

Meanwhile, the interaction of IgE with FcεRII is involved with several key processes associated with the type 2 immune response, including the regulation of IgE synthesis and allergen transcytosis [37]. Here, epithelial FcεRII contributes to the transport of IgE–allergen immune complexes across the airway epithelium to facilitate allergen sampling [37]. IgE–allergen complexes can be internalized by FcεRII on antigen-presenting cells, leading to allergen processing and presentation on the cell surface to activate T cells, producing additional IgE and amplifying the allergic inflammatory response [37]. Similarly, in B cells, the cross-linking of allergen-bound IgE to FcεRII leads to the internalization, processing, and presentation of antigens to T cells, leading to T cell activation and T cell–mediated allergic inflammation [38]. In response to the binding of IgE to FcεRII on B cells that are currently producing IgE, synthesis can be upregulated or downregulated, depending on the specific form of FcεRII [37].

As described, there are several causative factors of the type 2 inflammatory pathway (allergen exposure, *S. aureus* superantigen exposure, epithelial dysfunction leading to innate immunity) implicated in upper and lower airway diseases, and several opportunities for positive feedback to enhance the inflammatory response. At the heart of this inflammatory process lies the key mediator: IgE. Understanding the role of IgE in the pathobiology of each disease unifies the upper and lower airways.

## Upper Airway Disease

### CRSwNP

The long-lasting mucosal inflammation associated with CRSwNP is often Th2 derived and eosinophilic in nature, marked by highly elevated local IgE levels [1]. This elevated local IgE production may be the result of stimulation by extrinsic allergens. However, patients with non-allergic disease also exhibit elevated IgE levels, suggesting that other pathways may contribute to this inflammatory process [1]. Superantigens from viral or bacterial microorganisms can directly activate B cell cytokine release, leading to increased levels of IL-5 and eosinophils [39]. In response to superantigen exposure and subsequent epithelial damage, epithelial cell release alarmins (IL-25, IL-23, and TSLP), which amplify the type 2 immune response by activating ILC2 cells to produce additional IgE [24, 33]. One of the most common contributors to these superantigens is *S. aureus*, which has been identified in the NP tissue of 63.6% of patients with CRSwNP compared with 33% of controls. Importantly, these observations were independent of serum total or specific IgE levels [40].

One additional trigger of this inflammatory cascade leading to nasal polyposis is through exposure to fungi. Indeed, allergic fungal sinusitis represents a subset of CRSwNP in which patients exhibit eosinophilic mucus in the sinuses, IgE-mediated allergy to fungus, and the presence of fungus in the sinus mucus [41]. In these patients, a localized mucosal allergic response including local IgE synthesis mediates the inflammatory response, ultimately leading to nasal polyposis [41].

As part of the inflammatory response associated with nasal polyposis, activation of eosinophils, in addition to basophils and some T cells, leads to the overproduction of the galectin-10 protein and subsequent auto-crystallization, forming what is known as Charcot-Leyden crystals. These crystals have been observed in the mucosa and mucus of patients with CRSwNP, particularly in more severe cases [42]. Charcot-Leyden crystals can stimulate the innate immune response or adaptive immunity in the presence of allergens, leading to increased IgE production, enhancing the type 2 inflammatory response [42, 43]. Additionally, the presence of Charcot-Leyden crystals has been linked to epithelial recruitment of neutrophils, leading to persistent, severe airway disease, which is non-responsive to current therapies [42].

Unsurprisingly, similarities in disease pathology contribute to considerable overlap between patients with CRSwNP and asthma. Between 30 and 70% of patients with CRSwNP have comorbid asthma, and ~ 70% of patients with asthma have an elevated risk of CRSwNP [44–46]. In patients with CRSwNP, elevated total IgE levels are predictive of asthma comorbidity [23]. The upper and lower airways share many of the same histological structures, such as the basement membrane, lamina propria, and ciliary epithelium [47]. CRSwNP and asthma often present together [47]. Further, many of the prominent underlying pathological features of CRSwNP, including high expression levels of type 2 cytokines (IL-4, IL-5, and IL-13, among others) and elevated IgE, are also key contributors to the pathology of asthma, as described below [47]. Moreover, the polyclonal IgE produced following *S. aureus* superantigen exposure can contribute to the persistent type 2 inflammation associated with CRSwNP through continuous mast cell activation, and the elevated specific polyclonal IgE to *S. aureus* enterotoxins in the serum of these patients is a known risk factor for asthma severity [23].

### AERD

AERD, also called non-steroidal anti-inflammatory drug (NSAID)-exacerbated respiratory disease (N-ERD), is a form of asthma defined by three key factors: severe asthma, recurrent and severe NP, and acute respiratory response to NSAIDs [48]. Hallmarks of the disease include the overproduction of leukotrienes and prostaglandin D2, which are involved in the inflammatory and immune response pathways [49, 50]. Leukotrienes are potent bronchoconstrictors responsible for many of the symptoms in AERD, while prostaglandin D2 induces vasodilation and increases vascular permeability [24].

Although many patients with AERD have comorbid allergy, AERD also often occurs in non-allergic hosts with elevated eosinophilic inflammation and is sometimes associated with elevated total serum IgE, indicating that a pathway distinct from the antigen-specific type 2 allergic response is likely involved [51]. In one proposed disease mechanism, leukotriene-induced IL-33 expression activates the release of cytokines (IL-5 and IL-13) by ILC2 cells, leading to IgE production, activation, and degranulation of mast cells, and, ultimately, the eosinophilic inflammation commonly associated with AERD [51].

Similarly to other IgE-mediated airway diseases, the presence of specific IgE to *S. aureus* superantigens is observed in the local nasal tissue of patients with AERD [52]. It is suggested that this specific local IgE plays a role in modifying the severity of airway inflammation in patients with AERD [52]. This is in contrast to the limited reports linking serum-specific *S. aureus* IgE to AERD, emphasizing the role of local IgE in disease [52].

### Allergic Rhinitis

AR is defined as rhinitis or rhinoconjunctivitis where symptoms, including nasal congestion, nasal itch, rhinorrhea, and sneezing, present following exposure to an aeroallergen, most commonly dander, mold, pollen, or residues from cockroaches and dust mites, with evidence of specific IgE to that allergen detected by SPT or serum-specific IgE testing [53]. In patients with AR, allergen exposure leads to a rise in local and systemic specific IgE production [1]. Observed elevation in serum IgE in these patients is believed to be due to spillover from the affected organ, rather than being produced in the blood [1]. Upon allergen exposure, the IgE-mediated type 2 inflammatory cascade is initiated, as previously described [1]. Additionally, the IgE-mediated activation of airway epithelial cells can further amplify the allergic reaction through the innate immune response [1]. Studies have shown that the disease severity of AR can be modified by environmental factors, including temperature, humidity, and air pollution. AR also leads to a higher colonization rate of *S. aureus* and superantigen exposure, leading to increased Th2 cytokine production and inflammation and worsening disease severity [54].

### Non-allergic Rhinitis

A subset of patients with rhinitis are classified as having NAR, accounting for roughly 25% of all patients with rhinitis [55]. In these patients, no systemic markers of allergy, such as elevated antigen-specific IgE, are detected, despite the presence of rhinitis symptoms [1]. NAR can be induced by environmental irritants, such as change in weather, barometric pressure, temperature, or irritants, which cause abnormal vasomotor responses leading to mucus production and congestion. One common contributor to the pathophysiology of NAR is endonasal infections, which, along with exposure to exogenous irritants, may trigger mucosal inflammation through activation of T cell–mediated delayed-type hypersensitivity and activation of IgE-bearing mast cells via Ig-free light chains (FLCs) [1]. FLCs are a byproduct of receptor revision, wherein an ~ 10–40% excess of Ig light chains is produced versus the heavy-chain variant [56]. These FLCs have conventionally been thought of as inconsequential, contributing neither to specific receptor binding nor complement activation [56, 57]. More recently, however, these FLCs have been believed to serve as an alternative for IgE in the inflammatory pathway in several disorders by inducing hypersensitivity through mast cell activation. This mechanism has been implicated in both AR and NAR [1].

A further classification of rhinitis, local allergic rhinitis (LAR), describes the onset of disease symptoms with a localized allergic response in the nasal mucosa similar to that of AR; however, diagnostic tests, including SPT or serum IgE, are negative [58]. Despite negative systemic allergic testing, it is suspected that some patients with idiopathic rhinitis produce local specific IgE, indicating the presence of allergic disease [59]. This is further supported by evidence of the presence of Th2 inflammatory pathway components within the local tissue of patients with AR [60–63], suggesting the possibility of localized IgE synthesis and secretion [1]. Of note, asthma is a common comorbidity of rhinitis, regardless of allergic status, further suggesting a link between upper and lower airway disease [64].

## Lower Airway Disease

Asthma comprises a heterogeneous spectrum of lower airway diseases, rather than a single disease. Most commonly, asthma is defined by allergy status based on whether the clinical symptoms were precipitated by exposure to a common aeroallergen, as confirmed by a positive SPT or serum-specific IgE [65].

### Allergic Asthma

It is widely accepted that in response to an allergen exposure, susceptible patients experience elevated local specific clonal IgE, which is believed to be the cause of allergic airway inflammation in asthma through activation of the type 2 inflammatory cascade leading to eosinophilia [33, 66]. In support of this, type 2–high asthma is characterized by elevated levels of IgE and Th2 cytokines, including IL-4, IL-5, and IL-13 [66]. As previously described, these factors are key to the cyclic amplification of IgE production and subsequent inflammation associated with disease.

### Non-allergic Asthma

There are a considerable number of classifications of non-allergic asthma, making it more difficult to diagnose and manage [67, 68]. The variety of asthma phenotypes includes early onset, eosinophilic, obese, and neutrophilic [68]. Early-onset allergic asthma is typically most prevalent in childhood and early adulthood, with a switch toward non-allergic asthma in adulthood [24]. However, across these phenotypes, there is considerable overlap, and they are not mutually exclusive [68]. Disease presentation tends to be more severe in patients with non-allergic asthma versus allergic asthma and can present highly heterogeneously in terms of eosinophilia, fixed airflow limitation, association with chronic rhinosinusitis, and presence of neutrophilic inflammation [67]. Typically, patient age, age at onset, and female to male ratio are higher in patients with non-allergic asthma versus allergic asthma [65].

Despite this variability in non-allergic asthma subtypes, many of the underlying characteristics are similar to those in allergic asthma, with the same treatment approaches suggested for both [67]. These similarities include increased activation of the type 2 inflammatory cascade, as evidenced by elevated levels of IL-4, IL-5, and IL-13; the presence of eosinophilic inflammation, elevated local IgE, and airway remodeling, including epithelial denudation; and thickening of the basement membrane and bronchial smooth muscle, which is said to be indistinguishable between allergic and non-allergic asthma subtypes [69, 70]. Although a biomarker for early-onset allergic asthma, IgE levels are often elevated in non-allergic late-onset asthma, but this IgE is often polyclonal and, as in CRSwNP, attributed to *S. aureus* enterotoxins [13].

### Other Lower Airway Diseases

Additional diseases of the lower airway have been linked to IgE, namely allergic bronchopulmonary aspergillosis (ABPA) [71]. ABPA is a distinct endotype of allergic asthma characterized by markedly elevated IgE levels, allergic sensitization to *Aspergillus*, and mucus plugging. Inhalation of *Aspergillus* spores has been shown to cause ABPA in allergic individuals [71]. Repeated inhalation primarily elicits a type 1 allergic response in these patients, though type 3 and type 4 reactions also occur [71]. Exposure induces a polyclonal antibody response, leading to elevated total IgE levels as well as *Aspergillus*-specific IgE and IgG [71].

## Diagnostic Value of IgE

The central effector role of IgE in the type 2 inflammatory pathway across various upper and lower airway diseases supports the utility of IgE as a diagnostic marker. Proper testing is required to determine whether a symptomatic individual’s serum versus local IgE levels are elevated in order to provide an accurate diagnosis, because these details may directly impact the categorization of disease [1].

### Testing for Allergic Disease

The current diagnostic paradigm for upper and lower airway disease focuses on first differentiating between allergic and non-allergic disease. Symptomatic patients are initially assessed for patient history, with follow-up diagnostic testing geared toward identifying the presence of allergy [72, 73] through assessment of specific IgE levels, rather than local production [74].

An SPT represents a fairly common, relatively inexpensive test that provides immediate results and often serves as the first choice in confirmatory allergic testing [72, 74]. Despite its utility in identifying specific allergens in IgE-mediated allergic disease, the SPT has a few inherent limitations. SPT fails to examine the local production of IgE, such as in the nasal mucosa or lungs. In patients with only local IgE production, a SPT will yield a negative result, suggesting the absence of an allergic component [1]. Additionally, the use of SPT is highly variable by location, with a distinct lack of standardization in testing practices. Moreover, the use of allergy medications, including antihistamines, is known to interfere with the results and interpretation of SPT [75]. Scoring the SPT relies on the interpretation of a patient’s response in relation to their symptoms, which requires an experienced physician [76]. Ultimately, the utility of an SPT is bound by the specific panel of allergens used [76]. Often, a panel of eight to ten of the most common, locally relevant allergens is capable of identifying allergy in many patients, though a larger allergen panel may be required to fully assess a population [76].

Serum testing identifies the level of specific IgE in the blood of patients to specific selected allergens, and can be seen as complementary to SPT [77, 78]. Overall, serum-specific IgE testing is advantageous in that it can be performed in patients with skin disease and those who are receiving allergy medications such as antihistamines, which could interfere with SPT; it can identify sensitivity to potential cross-reacting allergens; and it eliminates the possibility of systemic reactions [75, 77]. However, these tests also come with their own limitations. First, they are time consuming and require a high volume of allergens and a blood sample to complete [79]. There is also currently no standardized reporting established for serum-specific IgE results, which can contribute to variability between tests [77]. Further, this form of testing may be more expensive than SPT methods, preventing its routine use [77]. The results obtained from serum-specific IgE testing may not correlate well with other testing methodologies, and may not be fully informative of clinical status, including disease severity, presence of tolerance through blocking IgG4 antibodies, or total IgE levels [77].

For patients in whom diagnostic uncertainty remains after routine methodologies are exhausted, more expansive testing, such as an immuno-solid-phase allergen chip (ISAC), may be performed. ISAC tests for specific IgE against multiple allergen components using a multiplex assay [74]. As ISAC testing assesses serum-specific IgE, it does not provide information regarding local IgE levels [74]. This methodology is highly expensive, so is often reserved until after other tests are performed, but it may yield more conclusive results [74]. Moreover, ISAC test results should be examined carefully because the associated benefit of testing multiple allergen components can inadvertently identify allergic sensitizations that are irrelevant to a patient or that do not contribute to symptoms [80].

Taken together, routine IgE testing methodologies come with their own advantages, yet they are also impacted by numerous practical limitations [74, 76–79, 81]. Additionally, these routine allergy testing methodologies must be further confirmed by the presence of symptoms or through allergen provocation assessments if a patient’s clinical history and sensitization disagree or are unavailable [82]. It is important to note, however, that many upper and lower airway diseases only present with local IgE increases rather than an allergic response, which cannot be diagnosed by routine SPT or elevated serum IgE methodologies. These situations require additional, localized testing to determine the role of IgE mediation.

### Testing for Local IgE: Unmet Need

As described, the current diagnostic paradigm in patients presenting symptoms of upper or lower airway disease relies on the use of routine allergy tests, which examine circulating specific or total IgE to identify the presence of an allergic component of the disease. In many upper and lower airway diseases, however, it is local IgE activity that drives symptomology through the type 2 inflammatory pathway [1, 72, 73].

After initial suspicion of asthma from patient history, routine allergic testing methodologies are often the first employed [67]. However, total IgE levels of patients with asthma often fall within the normal range, whereas other patients without asthma may present with elevated IgE outside of this range [83]. Additionally, patients with non-allergic asthma may only present with a localized polyclonal IgE response [13], despite similarities in underlying type 2 inflammatory disease pathobiology [67]. Failure to identify a specific allergen by conventional means does not indicate the absence of IgE-mediated asthma, because a local reaction may be the cause of symptomology [67].

Traditionally, the distinction between AR and NAR is based on an SPT and serum IgE analysis [1]. However, in the subgroup of patients with LAR, accumulating evidence suggests that local specific IgE production may occur in response to allergen exposure despite negative systemic tests [1]. Diagnostic tools in patients with rhinitis often fail to distinguish AR, NAR, and LAR because the measurement of local IgE by means of nasal provocation testing or nasal cytology is typically not performed in patients with rhinitis outside of clinical research, and its use in the routine evaluation of rhinitis is not recognized [1, 84]. Therefore, NAR is typically diagnosed by exclusion, because this group of patients has a poorly defined pathogenesis [1]. Some patients with rhinitis may exhibit elevated serum IgE levels; however, it has been shown that this rise is largely due to spillover from a local increase in IgE, further suggesting the need for screening at the local level [1].

In patients with CRSwNP, testing for specific and total IgE in the serum may predict asthma comorbidity and severity [23]. However, the high aeroallergen sensitivity and localized allergic response associated with CRSwNP has shown no differences in imaging, symptomatic severity, or disease recurrence in allergic versus non-allergic individuals [85]. In many patients with CRSwNP, the disease is highlighted by the presence of *S. aureus*–specific IgE in the local NP tissue [39], suggesting that the severe inflammatory response associated with contributing to CRSwNP stems from local polyclonal IgE involvement [1].

Together, these observations suggest that the role of IgE in some upper and lower airway diseases may be underestimated by initial testing of systemic total or specific IgE, further highlighting the need for diagnosis by a localized allergic response. In support of this, it has been suggested that in patients with a negative SPT or no evidence of elevated serum-specific IgE, additional testing should be utilized to identify patients with localized allergic responses [86, 87].

### Testing for Local IgE: Methodologies

The nasal allergen challenge has been used in combination with routine allergen tests to differentiate between patients with AR (positive SPT or serum-specific IgE), NAR (negative nasal allergy challenge), and LAR (negative serum-specific IgE/SPT, positive nasal allergy challenge) [86]. Despite this utility in AR, it has been demonstrated that the use of nasal provocation testing to determine allergic sensitization in patients with CRSwNP is limited, likely due to the polyclonality of local IgE- or IgG4-blocking activity in these patients [88]. Additionally, nasal allergen provocation tests or challenges must be performed under clinical supervision, are labor and resource intensive, have variable specificity, and are often stressful for the patient [81].

The most direct method for investigating the pathology of disease tissue, including accurate measurements of local IgE levels, involves the collection of tissue via biopsy [1]. Indeed, studies have demonstrated elevated polyclonal IgE, *S. aureus*–specific IgE, colonization with *S. aureus*, and tissue eosinophilia within the NP tissue of patients with CRSwNP [89]. However, because tissue biopsy can be invasive, it is only performed when a patient already requires surgical intervention [1]. Therefore, a tissue biopsy is of limited utility in routine diagnosis.

One technique used to sample local airway IgE is through the collection of nasal secretions by nasal lavage and absorption techniques, although sample collection from the lower airway via bronchosorption is more challenging [1]. Studies have suggested that cytokine detection in samples obtained by nasosorption and sputosorption techniques reflects lower airway eosinophilia, although these methodologies still require extensive validation [90]. These techniques are performed under the assumption that protein levels in these secretions are representative of those in the underlying mucosa [1]. In one study, patients from three trials were examined to determine the impact of four treatment methodologies (omalizumab, mepolizumab, methylprednisolone, and doxycycline) on periostin levels in either the nasal secretions or serum of patients with CRSwNP to determine its utility as a biomarker of response to treatment. Improvements in eosinophilic inflammation and clinical outcomes corresponded with periostin levels, indicating that treatment disrupts the inflammatory cascade at both local and systemic levels [91]. However, it is important to note that in measuring IgE levels there exists a disconnect between local and serum total IgE when treating with omalizumab, as omalizumab-IgE complexes form with an extended half-life, falsely increasing total IgE measures [91].

Another study pioneered the use of an allergen microarray chip in assessing levels of local specific IgE to house dust mites (HDM) in the nasal secretions of patients with AR. Here, total and specific IgE levels to HDM were measured in the serum and nasal secretions of patients with confirmed allergic sensitization to HDM using ImmunoCAP fluorescent enzyme immunoassay and customized microarray assays to determine whether these measurements correlated. Overall findings in this study suggested good sensitivity using the microarray approach, superior to that of ImmunoCAP fluorescent enzyme immunoassay, in the detection of local specific IgE to HDM in nasal secretions of patients with AR. This suggests the utility of microarray technology as a non-invasive alternative to measuring IgE levels in the nasal secretions of patients with AR [92]. Finally, the utility of the ISAC chip in assessing response to allergen-specific immunotherapy in patients with allergic sensitization to HDM has previously been examined. Here, the ISAC test was used to examine the protection exhibited by treatment on a variety of HDM allergens through analysis of patient serum and nasal secretions. Ultimately, it was found that patients with varying HDM allergic sensitization profiles responded differently to treatment, and therefore, stratifying patients by their specific sensitization profile may better guide treatment [93].

The basophil activation test serves as a useful ex vivo test of allergic response, in which basophils from whole blood or isolated peripheral blood mononuclear cells are exposed to allergens of interest, and the subsequent expression of basophil activation markers in response to exposure is measured by flow cytometry [81, 94]. Although the analysis of whole and isolated peripheral blood samples is more typically used in the detection of systemic allergen response, recent studies have shown that this technique may hold a utility as a non-invasive analysis of localized response [81]. In patients with LAR, the basophil activation test has demonstrated sensitivity sufficient to diagnose an IgE-mediated response where an SPT and serum-specific IgE testing were found to be negative [95]. Moreover, studies have shown that the use of antihistamines does not impact the results of the basophil activation test, and they therefore do not need to be discontinued before testing [78]. One potential drawback of these tests, however, is that they rely on the prior confirmation of an allergen of interest by SPT or measure of serum-specific IgE [94]. Additionally, there is currently limited standardization regarding the technique and interpretation of basophil activation test outcomes [78]. With these established, the basophil activation test serves as a promising methodology for determining IgE activity at both the local and systemic levels.

Although there are many available diagnostic tools to determine the role of IgE in disease, there is still room for improvement. Negative results on the most routinely used tests (SPT, serum testing for allergen-specific IgE) do not conclusively indicate the lack of an allergic response, and neither do they identify local innate immunity. However, more localized tests can be invasive, not broadly applicable, and are limited by the lack of routine use in practice. As technology and standardization of practice continue to improve, so too will the diagnosis of IgE-mediated disease. In the meantime, the use of a treatment that addresses the common, underlying factor in these diseases can serve to improve patient outcomes.

## Anti-IgE as an Effective Treatment

As previously described, the common underlying pathophysiology of many upper and lower airway diseases is suggestive of a continuous or “unified” airway [2, 3]. An IgE-mediated inflammatory process is the key driver, leading to epithelial injury and airway remodeling [2, 3]. Targeting this process with anti-IgE therapy is effective in both allergic and non-allergic diseases, expanding the prior notion of IgE involvement beyond allergic asthma [33]. The efficacy of the anti-IgE therapy omalizumab has led to its approval in the treatment of allergic asthma, severe AR, and CRSwNP in a variety of countries [96–98].

In upper airway disease, anti-IgE therapy for NP has shown significant improvements in both clinical and patient-reported outcomes [97]. Similarly, in patients with AR, anti-IgE therapy demonstrated safe control of symptoms, improved quality of life, minimized use of rescue medications, and prevented asthma exacerbations in patients with comorbid asthma [98–102]. In patients with CRSwNP, anti-IgE therapy led to reductions in inflammation as measured by imaging [103]. Patients with asthma with comorbidities from the PROSPERO and EXTRA asthma trials showed reductions in the number of exacerbations experienced comparable with patients with asthma without comorbidities, whereas improvements in forced expiratory volume in 1 s were greater in patients with comorbidities than without [104].

In patients with lower airway disease, similar efficacy has been demonstrated in response to anti-IgE therapy. In patients with moderate-to-severe allergic asthma inadequately controlled by medium- to high-dose inhaled corticosteroids, anti-IgE therapy demonstrated a significant reduction in asthma exacerbations [105]. Interestingly, patients whose asthma responded well to treatment from the SOLAR trial saw a significant likelihood of achieving improvements in comorbid rhinitis as well [106]. Finally, anti-IgE therapy has shown efficacy in a number of non-allergic asthma subtypes, including non-atopic asthma [107–110], severe eosinophilic asthma [111–113], AERD [114–119], and severe occupational asthma [120].

Taken together, the clinical efficacy of anti-IgE therapy across numerous airway diseases challenges the traditional allergen-centric definition of upper and lower airway disease. Rather than distinguishing between allergic/atopic versus non-allergic/non-atopic disease, it may be more appropriate to think of disease as responses driven by the type (i.e., monoclonal vs. polyclonal) and location (i.e., upper vs. lower airway) of IgE-mediated versus non–IgE-mediated disease (Table 1).

**Table 1:** Classification schemes of upper and lower airway disease

**Current allergy-dependent classification**	
**Allergic disease**	**Non-allergic disease**
Allergic asthma	Nasal polyposis
Allergic rhinitis (local or systemic)	AERD
ABPA	Non-allergic rhinitis
	Non-allergic asthma
**Proposed IgE-dependent classification**	
**IgE-mediated disease**	**Non–IgE-mediated disease**
Th2 asthma (allergic, EIA, AERD, late-onset eosinophilic)	Some non-allergic rhinitis subgroups
ABPA	CRSsNP
Allergic rhinitis	Non-allergic asthma:
Some non-allergic rhinitis subgroups	•Neutrophilic
CRSwNP (nasal polyposis)	•Obesity associated
	•Smoking associated
	•Very late onset
	• Environmental asthma due to chemical or pollution exposure

*ABPA* allergic bronchopulmonary aspergillosis, *AERD* aspirin-exacerbated respiratory disease, *CRSsNP* chronic rhinosinusitis without nasal polyps, *CRSwNP* chronic rhinosinusitis with nasal polyps, *EIA* exercise-induced asthma, *IgE* immunoglobulin E, *Th2* T helper 2

In defining disease by the role of IgE (e.g., IgE mediated vs. non–IgE mediated), physicians can diagnose based on local or systemic IgE involvement as the underlying cause of disease and effectively treat at the source. Prompt reduction in IgE levels can disrupt the cascade of inflammation, including eosinophilic involvement, by using a targeted therapeutic, regardless of allergic status. Because IgE mediates the inflammatory response underlying many upper and lower airway diseases, halting its activity quickly and completely may lead to efficient symptom reduction in patients [96]. Targeting IgE directly using the anti-IgE antibody omalizumab provides a prompt reduction in free IgE levels, with patients with allergic asthma reporting > 95% reduction within days after starting therapy [121]. This is in comparison with other treatment options targeting other drivers of type 2 inflammation, such as mepolizumab, which blocks IL-5 signaling and has no effect on IgE levels in patients with severe asthma [122], and dupilumab, which blocks IL-4 and IL-13 signaling [123] and gradually decreases serum total IgE levels (70% reduction from baseline at 52 weeks) in patients with allergic asthma [124]. The differences in timing and magnitude of IgE reduction between treatment options are likely due to their different mechanisms of action, with omalizumab targeting IgE directly.

For omalizumab, additional studies have investigated the complexities of treating patients with anti-IgE. Although serum total IgE levels are useful, they are not predictive of clinical response to omalizumab [97, 125–127]. Additionally, serum total IgE levels are known to increase following omalizumab treatment initiation due to IgE–anti-IgE complexes forming, and it is unclear how this can affect dosing [128]. Further, both total IgE and specific-allergen IgE have been associated with omalizumab treatment response in asthma [129]. These findings further emphasize the need to accurately assess different IgE parameters (for example, serum IgE, specific IgE, local IgE) to provide an accurate diagnosis and guide choice of treatment with anti-IgE drugs, such as omalizumab, or with other treatments that lower IgE levels, such as dupilumab.

## Summary

There is considerable evidence supporting the role of IgE in many upper and lower airway diseases as the underlying cause of damaging inflammation independent of allergy [1, 33]. Further, this evidence suggests that in many of these airway diseases, there exists some degree of both local allergic IgE production and a local non-allergic IgE response through innate immunity or superantigens [8]. This convergence on the unifying component in the pathobiology of these diseases highlights the utility of IgE as a diagnostic tool. However, current methodologies focus primarily on distinguishing allergic and non-allergic disease by measuring systemic IgE via SPT or serum-specific IgE testing, rather than investigating the disease-modifying role of local IgE.

This diagnostic paradigm poses a particular problem in the treatment of diseases lacking an allergic component, because evidence has accumulated in support of the efficacy of anti-IgE therapeutics in treating both upper and lower airway disease, regardless of allergy status or total IgE levels. The routine testing for the presence of specific IgE to diagnose allergy status rather than assessing local IgE levels prevents physicians from identifying the disease for which local IgE causes symptomology and which will respond to anti-IgE therapy. Similarly, when utilizing anti-IgE therapy, evidence suggests clinical efficacy regardless of total IgE levels.

Rather than distinguishing between allergic/atopic versus non-allergic/non-atopic disease, it may be more appropriate to think of disease as responses driven by the type (i.e., monoclonal vs. polyclonal) and location (i.e., upper vs. lower airway) of IgE-mediated versus non–IgE-mediated disease. When interpreting test results, it is important to consider the involvement and diagnosis of local as well as systemic IgE. Doing so would allow for the effective, targeted treatment of IgE-mediated upper and lower airway disease by addressing the root cause of the disease, regardless of allergic status.
